# The Heterocyst-Specific Small RNA NsiR1 Regulates the Commitment to Differentiation in Nostoc

**DOI:** 10.1128/spectrum.02274-21

**Published:** 2022-03-01

**Authors:** Manuel Brenes-Álvarez, Agustín Vioque, Alicia M. Muro-Pastor

**Affiliations:** a Instituto de Bioquímica Vegetal y Fotosíntesis, Consejo Superior de Investigaciones Científicas and Universidad de Sevilla, Seville, Spain; Forschungszentrum Jülich GmbH

**Keywords:** *Anabaena*, regulatory RNAs, cyanobacteria, heterocyst differentiation, small RNAs

## Abstract

Heterocysts are specialized cells that filamentous cyanobacteria differentiate for the fixation of atmospheric nitrogen when other nitrogen sources are not available. Heterocyst differentiation at semiregular intervals along the filaments requires complex structural and metabolic changes that are under the control of the master transcriptional regulator HetR. NsiR1 (nitrogen stress-induced RNA 1) is a HetR-dependent noncoding RNA that is expressed from multiple chromosomal copies, some identical, some slightly divergent in sequence, specifically in heterocysts from very early stages of differentiation. We have previously shown that NsiR1 inhibits translation of the overlapping *hetF* mRNA by an antisense mechanism. Here, we identify *alr3234,* a *hetP-*like gene involved in the regulation of commitment (point of no return) to heterocyst differentiation, as a target of NsiR1. A strain overexpressing one of the identical copies of NsiR1 commits to heterocyst development earlier than the wild type. The posttranscriptional regulation exerted by NsiR1 on the expression of two genes involved in heterocyst differentiation and commitment, *hetF* and *alr3234*, adds a new level of complexity to the network of transcriptional regulation and protein-protein interactions that participate in heterocyst differentiation.

**IMPORTANCE** Heterocysts are nitrogen-fixing specialized cells that appear at semiregular intervals along cyanobacterial filaments upon nitrogen starvation. The differentiation and patterning of heterocysts is a model for the study of cell differentiation in multicellular prokaryotes. The regulation of differentiation, which is only partially understood, includes transcriptional changes, factor diffusion between cells, and protein-protein interactions. This work describes the identification of a novel target for NsiR1, a small RNA (sRNA) encoded in multiple slightly divergent copies, and shows how different copies of “sibling” sRNAs regulate the expression of different targets involved in one of the few examples of a differentiation process in prokaryotes.

## INTRODUCTION

Cyanobacteria are the only bacteria that perform oxygenic photosynthesis. They are essential primary producers and play an important role in the carbon and nitrogen biogeochemical cycles of the biosphere. Some filamentous cyanobacteria, such as Nostoc sp. strain PCC 7120 (also known as Anabaena sp. strain PCC 7120), have the capacity to differentiate a specialized cell type devoted to nitrogen fixation, the heterocyst. When nitrogen deprivation is encountered, heterocysts differentiate from vegetative cells in a semiregular pattern along the filaments. Heterocyst differentiation involves a complex developmental program (1–4) that is ultimately under the control of NtcA, the global regulator of nitrogen assimilation (5), but also under the control of HetR, a specific regulator of cell differentiation (6, 7). HetR is a DNA binding protein that positively regulates its own expression and the expression of other genes involved in heterocyst differentiation (8). The expression of *hetR* is regulated at several levels. For instance, *hetR* is transcribed from a complex promoter that includes transcriptional start sites (TSSs) specifically induced in cells becoming heterocysts (9, 10). The levels of HetR are additionally regulated by the protease HetF (11, 12), although the precise mechanism is currently unknown.

Because heterocysts are terminally differentiated nondividing cells, a key aspect of differentiation is the so-called commitment point, an irreversible stage of differentiation from which the process is no longer halted by the addition of combined nitrogen. Several genes related to the regulation of commitment in *Nostoc* sp. PCC 7120 have been identified, including *hetP* (13) and its homologs *asl1930*, *alr2902*, and *alr3234* (14). The proteins Asl1930, Alr2902, and Alr3234 are thought to interact with HetR and with each other, regulating commitment through protein-protein interactions (14). Mutant strains with genes *alr3234* or *asl1930* deleted reach the commitment point earlier than the wild-type strain, suggesting that Alr3234 and Asl1930 might function as negative regulators, delaying commitment (14).

Noncoding small RNAs (sRNAs) are important regulators of gene expression in bacteria, including cyanobacteria (15, 16). They bind to their target mRNAs, most frequently inhibiting translation, but a large diversity of alternative regulatory mechanisms operated by sRNAs is known (17). They regulate various processes related to stress response or adaptation to a changing environment, including cell differentiation. In Bacillus, the expression of several sRNAs is restricted to the forespore (18–21), and in heterocyst-forming cyanobacteria, sRNAs have been identified as having heterocyst-specific expression (22–24) or affecting heterocyst differentiation (25).

**FIG 1:**
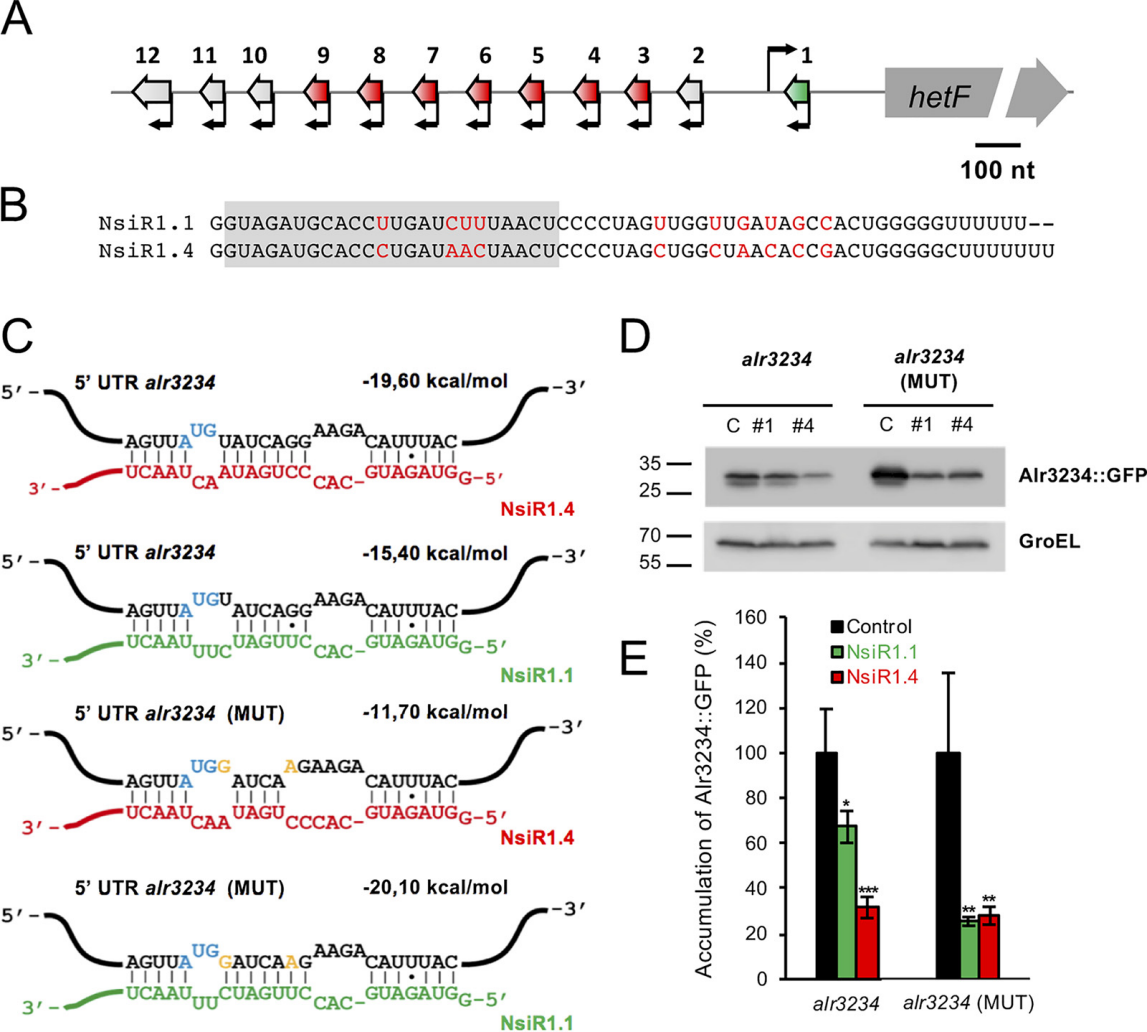
NsiR1 versions and their interaction with the *alr3234* 5′ UTR in E. coli. (A) The genomic region of *Nostoc* sp. PCC 7120 encoding NsiR1. Identical repeats of *nsiR1* (3 to 9, e.g., *nsiR1.4*) are shown as red arrows. Repeats of *nsiR1* with some sequence divergence are shown in light gray except for repeat 1 (*nsiR1.1*), antisense to *hetF*, which is shown in green. (B) Comparison of the sequences of NsiR1.1 and NsiR1.4. Nucleotide differences are highlighted in red. The region involved in the interaction with *alr3234* mRNA is shaded. (C) Predicted interactions between NsiR1.1 or NsiR1.4 and two versions of the 5′ UTR of *alr3234*. Nucleotides corresponding to NsiR1.1, NsiR1.4, the start codon of *alr3234*, and mutations introduced into the *alr3234* (MUT) 5′ UTR version are shown in green, red, blue, and orange, respectively. (D) Accumulation of the GFP protein in E. coli cells bearing several combinations of plasmids expressing different versions of *alr3234*::sf*gfp* and control RNA (C), NsiR1.1 (#1), or NsiR1.4 (#4). Western blot analyses were performed using antibodies against GFP or GroEL (loading control). (E) Quantification of Western blots. The data are presented as the mean values ± standard deviations of the GFP signal, normalized to the GroEL signal, from three independent experiments like the one shown in panel D with each combination of plasmids. The mean accumulation of GFP in the control strain in each experiment is used as 100%. *t* test: *, *P* < 0.05; **, *P* < 0.01; ***, *P* < 0.001.

NsiR1 (nitrogen stress-induced RNA 1) is an sRNA of about 60 nucleotides encoded in 12 tandem copies in the region upstream from *hetF* in Nostoc sp. PCC 7120 (Fig. 1A) (22). The different copies are transcribed from individual promoters that contain the DIF1 sequence motif associated with heterocyst-specific transcription (10, 23), and thus, NsiR1 is expressed specifically at an early stage of heterocyst differentiation (26). The seven central copies *nsiR1.3* to *nsiR1.9* (hereinafter referred to as *nsiR1.4*) are identical, whereas the other five copies, located at the periphery of the array, have some sequence divergence. NsiR1.1 is transcribed antisense to the 5′ untranslated region (UTR) of the *hetF* transcript and has been shown to inhibit the translation of this transcript in heterocysts, therefore affecting HetR accumulation (27). In addition to its putative protease activity, HetF is an interacting member of the division machinery under certain growth conditions (28), which may explain the observation of enlarged cells in *hetF* mutants (12).

Although the seven identical copies (*nsiR1.4*) are very similar to *nsiR1.1* (Fig. 1B) and can also partially regulate the expression of *hetF* (27), they could have additional targets in *Nostoc* sp. PCC 7120. In fact, Nostoc strains constitutively overexpressing *nsiR1.1* or *nsiR1.4* have slightly different phenotypes (27), suggesting that different versions of NsiR1 could have exclusive targets. Here, we identify the *hetP* homolog *alr3234* as an additional negatively regulated target of NsiR1. Therefore, sequence divergence between the different versions of NsiR1 allows the regulation of diverse aspects of heterocyst differentiation through their effects on the amounts of the HetF protease (influencing HetR accumulation and possibly nondivision of heterocysts) and protein Alr3234 (influencing commitment to differentiation).

## RESULTS

### Strain OE_NsiR1.4, overexpressing NsiR1.4, differentiates heterocysts in plates with nitrate as the nitrogen source.

The observation that constitutive overexpression of NsiR1.4, and not NsiR1.1, leads to a slightly slower growth in medium with nitrate as the nitrogen source (27) prompted us to analyze the filaments in more detail. We detected that strain OE_NsiR1.4, overexpressing NsiR1.4, differentiates patterned heterocysts in plates with nitrate as the nitrogen source (Fig. S1A in the supplemental material), a condition under which heterocyst differentiation should not take place, and this unexpected differentiation process was not due to lack of nitrate assimilation (Fig. S1B). Although strain OE_NsiR1.4 showed about half of the nitrate and nitrite reductase activities of the control OE_C strain, a previously described mutant strain with a similar reduction in nitrite reductase activity does not differentiate heterocysts (29), therefore suggesting that the heterocyst differentiation observed in strain OE_NsiR1.4 in the presence of nitrate is not due to a defect in nitrate assimilation.

### NsiR1.4 interacts with the 5′ UTR of *alr3234* in Escherichia coli.

A previous computational prediction (22) identified *alr3234* as a possible target of NsiR1.4 (transcribed from the seven identical copies of *nsiR1*). The predicted interaction of NsiR1.4 was located in the translation initiation region of the *alr3234* mRNA (Fig. 1C). IntaRNA (30) also predicted an interaction between NsiR1.1 (encoded by the slightly divergent *nsiR1* located upstream from the *hetF* gene) and the 5′ UTR of *alr3234*, but the hybridization energy was much lower (Fig. 1C).

We tested the interaction of the two slightly different sRNAs, NsiR1.4 and NsiR1.1, with the *alr3234* 5′ UTR using a heterologous reporter system in E. coli (31) in which the 5′ UTR of *alr3234* plus 60 nucleotides of its coding region were fused to the gene sf*gfp*, which encodes superfolder GFP (sfGFP). We coexpressed in E. coli the *alr3234*::sf*gfp* fusion and NsiR1.4, NsiR1.1, or a control unrelated RNA corresponding to the *T1* terminator of the *rrnB* gene of E. coli. For these experiments, both the *alr3234*::sf*gfp* translational fusion and the different versions of the RNAs were constitutively transcribed from the P_LtetO_ promoter (*alr3234*::sf*gfp* fusions) or the P_LlacO_ promoter (NsiR1.4, NsiR1.1, or the control RNA).

Cells carrying the *alr3234*::sf*gfp* fusion had very low fluorescence (not shown), preventing fluorimetric quantification, but we could instead measure the accumulation of Alr3234::sfGFP in E. coli by Western blotting using anti-GFP antibodies (Fig. 1D). The accumulation of Alr3234::sfGFP translated from the *alr3234* 5′ UTR decreased approximately 70% with respect to its accumulation in the control strain when NsiR1.4 was coexpressed and approximately 30% when NsiR1.1 was coexpressed (Fig. 1D and E). We then prepared an *alr3234*::sf*gfp* fusion containing two point mutations that, according to the predictions, would result in better binding to NsiR1.1, *alr3234* (MUT) 5′ UTR (Fig. 1C). In this case, the accumulation of Alr3234::sfGFP decreased similarly when this mRNA was coexpressed with either NsiR1.1 or NsiR1.4 (Fig. 1D and E). In the presence of the control RNA, the Alr3234::sfGFP accumulated in E. coli at higher levels than did the wild-type version. This could be due to the nucleotide changes introduced in the *alr3234* (MUT) 5′ UTR, which resulted in the replacement of tyrosine with aspartic acid at the first position after the initial methionine in the coded protein. According to the N-end rule (32), this change could result in a more stable protein.

**FIG 2:**
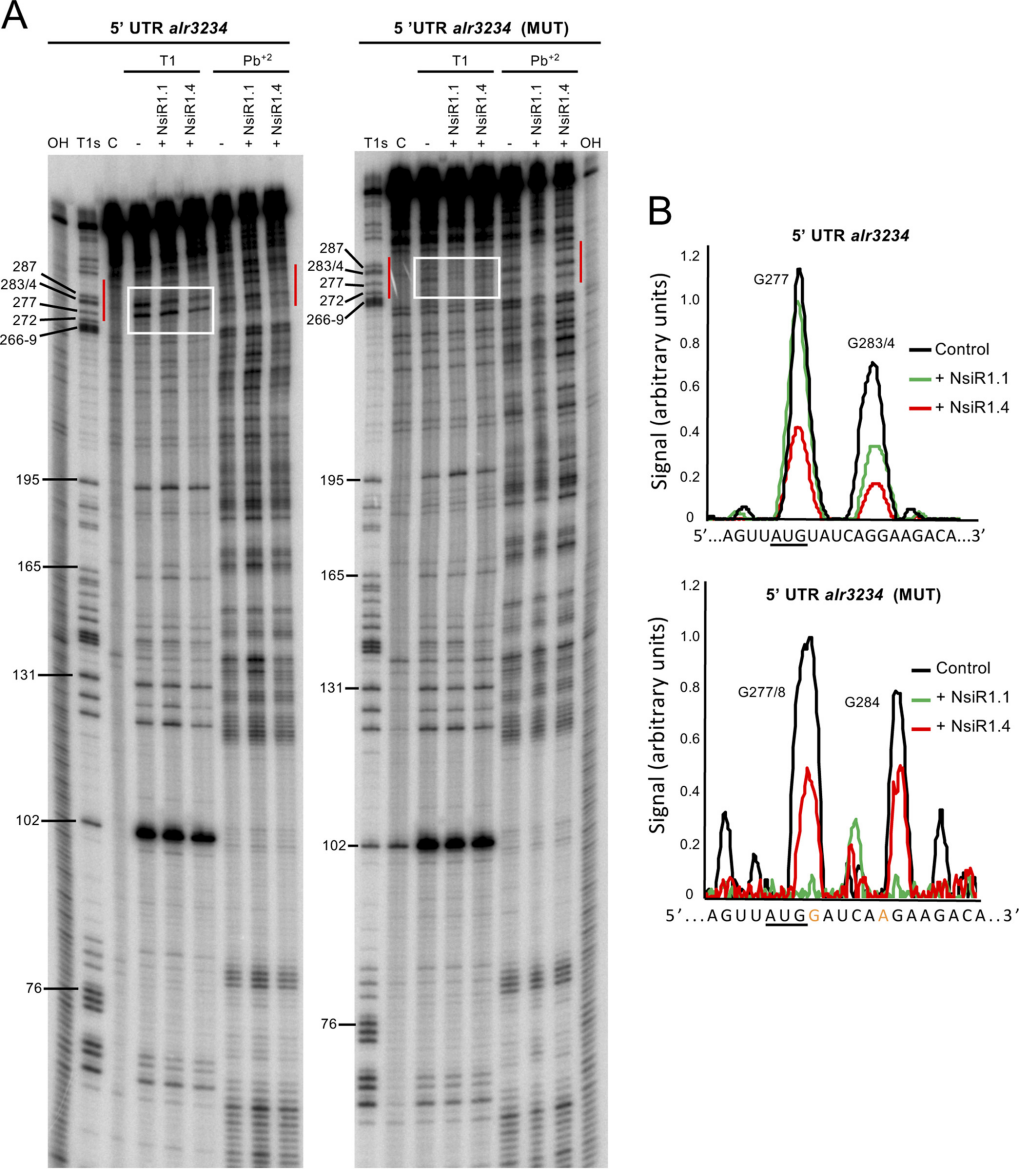
Interaction of NsiR1 with the *alr3234* 5′ UTR *in vitro*. (A) RNase T1 and lead(II) acetate (Pb^+2^) footprinting of the interaction between NsiR1.1 or NsiR1.4 and the wild-type and (MUT) versions of the *alr3234* 5′ UTR. The protected areas are indicated by red vertical bars. The nucleotide positions of either version of the *alr3234* 5′ UTR are shown on the left. C, untreated control; OH, alkaline ladder; T1s, RNase T1 sequencing ladder. Lanes marked with a minus sign correspond to the treatment with T1 or Pb^+2^ in the absence of NsiR1.1 or NsiR1.4. (B) Quantification of the signals in the regions framed in white in the gels. The start codon is underlined. Nucleotides changed in the *alr3234* (MUT) 5′ UTR are highlighted in orange.

We also performed *in vitro* footprinting assays of the interaction of NsiR1.1 and NsiR1.4 with the wild-type version of the *alr3234* 5′ UTR or with the *alr3234* (MUT) 5′ UTR, which should interact more efficiently with NsiR1.1 than with NsiR1.4. The ^32^P-labeled *alr3234* 5′ UTR versions were incubated with unlabeled NsiR1.1 or NsiR1.4 and probed with RNase T1 or lead(II) acetate (Fig. 2). We only detected footprints in the predicted region when the *alr3234* 5′ UTR was combined with NsiR1.4 or when the *alr3234* (MUT) 5′ UTR was combined with NsiR1.1 (Fig. 2). Taken together, these results indicate that NsiR1.4 can bind to *alr3234* mRNA and regulate *alr3234* mRNA expression.

### NsiR1.4 regulates the expression of *alr3234* in *Nostoc*.

**FIG 3:**
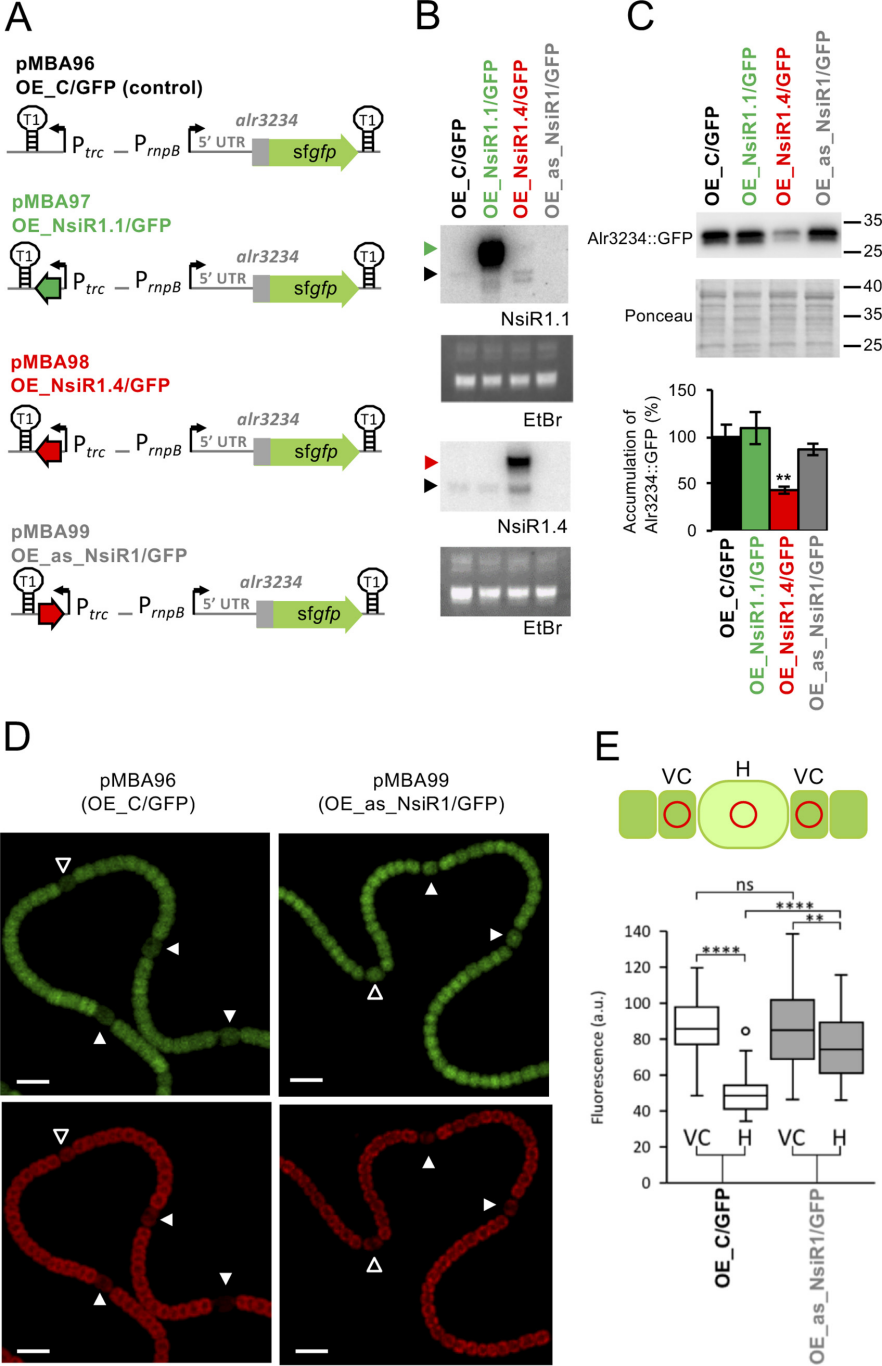
NsiR1.4 interacts with the 5′ UTR of *alr3234* in *Nostoc*. (A) Scheme of DNA fragments cloned in the plasmids used to generate *Nostoc* strains with altered levels of NsiR1.1 or NsiR1.4 in combination with the 5′ UTR of *alr3234* fused to sf*gf*p. Transcription start sites (bent arrows), *T1* terminators (stem-loops), promoters *trc* and *rnpB*, *nsiR1.1* (dark green arrow), *nsiR1.4* (red arrow), and the 5′ UTR of *alr3234* plus 60 nucleotides of its coding region fused to sf*gfp* are indicated. (B) Northern blot analyses using RNA extracted from the different strains 18 h after combined nitrogen removal hybridized with probes for NsiR1.1 (top) and NsiR1.4 (bottom). Ethidium bromide staining of the membranes (EtBr, bottom) is shown as a loading control. The samples contained 6 μg of total RNA. Endogenous NsiR1 (black triangle), the 6-nucleotide-longer NsiR1 constitutively expressed from the *trc* promoter, NsiR1.1 (green triangle), and NsiR1.4 (red triangle), are indicated. (C) Accumulation of Alr3234::sfGFP in the different strains 18 h after the removal of combined nitrogen. Western blot analyses were performed using 40-μg amounts of soluble fractions and antibodies against GFP (top). Signals were quantified with ImageLab software and normalized to Ponceau red staining (middle). The positions of the size markers (in kDa) are indicated on the right. Two technical replicates of three different clones of each strain were quantified. Values are expressed as percentages and standard deviations of the normalized amount of Alr3234::sfGFP with respect to the amount in the control strain (OE_C/GFP), which was considered 100% (bottom). *t* test: **, *P* < 0.01. (D) Confocal fluorescence images of filaments bearing plasmid pMBA96 or pMBA99 growing on top of nitrogen-free medium are shown for the green channel (GFP fluorescence) and red channel (autofluorescence). The loss of autofluorescence from photosynthetic pigments is a characteristic feature of heterocyst differentiation. Cells at a very early stage of differentiation are indicated with empty white triangles. Cells at a more advanced stage of differentiation and mature heterocysts are indicated with filled white triangles. Scale bars, 10 μm. (E) Quantification of the GFP fluorescence in heterocysts (H) and adjacent vegetative cells (VC). The expression of *alr3234*::sf*gfp* in heterocysts and vegetative cells was quantified by measuring the GFP fluorescence intensity in regions of interest (ROIs, red circles in the scheme) corresponding to 44 (OE_C/GFP) or 54 heterocysts (OE_as_NsiR1/GFP) and the two vegetative cells adjacent to each heterocyst. *t* test: **, *P* < 0.01; ****, *P* < 0.0001; ns, not significant.

To confirm the interactions tested in E. coli (Fig. 1) and *in vitro* (Fig. 2), we prepared *Nostoc* strains with altered levels of NsiR1 that, in addition, constitutively expressed the *alr3234*::sf*gfp* fusions analyzed in the E. coli system described above (Fig. 3A). Specifically, we prepared strains that overexpressed NsiR1.1, NsiR1.4, an antisense of NsiR1.4 (as_NsiR1) whose transcription leads to the depletion of NsiR1 (27), or an unrelated nonsense sRNA used as a control. It should be noted that, because we introduced a restriction site (6 bp) at the 5′ end of *nsiR1.1* or *nsiR1.4* to facilitate cloning, we could discriminate molecules of NsiR1.1 and NsiR1.4 transcribed from their native chromosomal promoters from molecules expressed from the *trc* promoter in the constructs introduced into *Nostoc* (Fig. 3B). Compared to the control strain OE_C/GFP, strain OE_NsiR1.4/GFP, overexpressing NsiR1.4 (Fig. 3B), had a significant decrease in the accumulation of Alr3234::sfGFP (Fig. 3C). This reduction was not observed in the strain OE_NsiR1.1/GFP, which overexpresses NsiR1.1 (Fig. 3B and C). These results are consistent with the different hybridization energies predicted for the interaction of NsiR1.1 and NsiR1.4 with the *alr3234* 5′ UTR (Fig. 1C) and confirm a functional divergence between these two versions of NsiR1.

The analysis described above involved the constitutive expression, in all cells of the filaments and irrespective of the nitrogen source, of both the *alr3234*::sf*gfp* fusion and the sRNA (NsiR1.1, NsiR1.4, or as_NsiR1). We then analyzed the accumulation of the constitutively expressed reporter *alr3234*::sf*gfp* in vegetative cells and heterocysts of nitrogen-fixing filaments by fluorescence microscopy in the strain depleted of NsiR1 (OE_as_NsiR1/GFP) in comparison to its accumulation in the control strain (OE_C/GFP), in which NsiR1.1 and NsiR1.4 are expressed from their native, heterocyst-specific promoters. The OE_C/GFP strain showed a strong decrease of fluorescence of the reporter Alr3234::sfGFP in those cells becoming heterocysts (compared with the fluorescence in the adjacent vegetative cells), even at a very early stage in the differentiation process (Fig. 3D and E). In contrast, the fluorescence in heterocysts of strain OE_as_NsiR1/GFP, depleted of NsiR1, was much less reduced than the fluorescence in the control strain OE_C/GFP. Thus, depletion of NsiR1 in the OE_as_NsiR1/GFP strain prevents the posttranscriptional downregulation of *alr3234* in heterocysts. The OE_as_NsiR1/GFP strain expresses the antisense to NsiR1 RNA constitutively in all cells, but vegetative cells of this strain have similar GFP fluorescence to vegetative cells of the control strain, indicating that the effect of depleting NsiR1 is restricted to heterocysts. This result supports the conclusion that the fluorescence reduction observed in heterocysts for the reporter Alr3234::sfGFP is specifically due to NsiR1, as NsiR1 is expressed exclusively in the heterocysts.

### Altered time of commitment to differentiate in strains overexpressing or lacking NsiR1.4.

**FIG 4:**
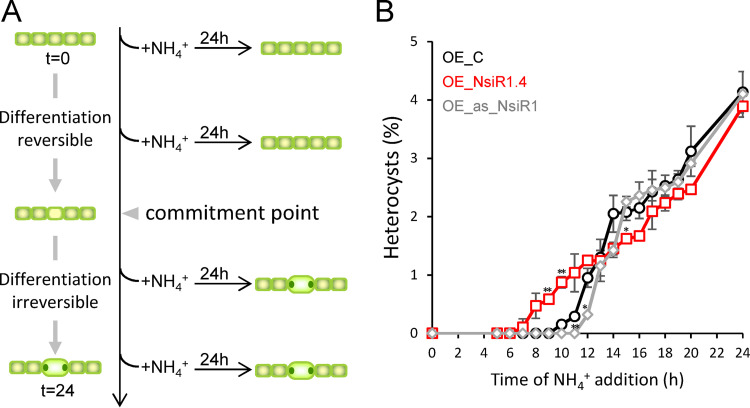
Determination of the time of heterocyst commitment in strains with altered levels of NsiR1. (A) Scheme of the experimental setup. After combined nitrogen removal (*t* = 0 h), ammonium was added to aliquots retired at different times and the frequency of heterocysts was determined 24 h after ammonium addition. If ammonium was added once the commitment point had been reached, the differentiation process was irreversible and heterocysts would differentiate despite the addition of ammonium. (B) Quantification of the numbers of heterocysts. Each data point represents the percentage of heterocysts observed 24 to 36 h after the addition of ammonium. For each strain, the data are presented as the mean values and standard deviations of two cultures from two independent clones. *t* test: *, *P* < 0.05; **, *P* < 0.01.

It has been described that in an *alr3234* null mutant, proheterocysts commit to differentiation earlier than in the wild-type strain (14). Commitment time is defined as the time after nitrogen removal at which heterocyst differentiation is no longer reversible upon the addition of ammonium to the medium. To determine possible changes in the commitment time among strains with altered levels of NsiR1, we carried out a heterocyst commitment assay in which ammonium was added at different time points after nitrogen removal and the final frequency of heterocysts in each sample was recorded (Fig. 4A). For this purpose, we used strains OE_C, OE_NsiR1.4, and OE_as_NsiR1 (27). While all three strains analyzed showed similar frequencies of heterocysts after 72 h of nitrogen removal (27), the heterocyst commitment assay showed that strain OE_NsiR1.4 (which overexpresses NsiR1.4 in all cells of the filaments, regardless of the nitrogen source) had an earlier onset of commitment than the control strain OE_C (Fig. 4B). While the first committed heterocysts appeared in the control strain after 9 h in the absence of combined nitrogen, in the strain overexpressing NsiR1.4, the first committed heterocysts appeared after 6 h in the absence of combined nitrogen. In contrast, a delay in commitment was observed in strain OE_as_NsiR1 (which is depleted of NsiR1), in which the first committed heterocysts appeared after 11 h of nitrogen deprivation. These results are consistent with posttranscriptional negative regulation of *alr3234* by NsiR1.4.

## DISCUSSION

Differentiation of heterocysts is a precisely regulated, complex process required to establish specific transcriptional programs for different cell types (vegetative cells, immature proheterocysts, and mature heterocysts). Heterocyst differentiation is triggered by mutual induction of the expression of the transcriptional regulator NtcA and the master regulator HetR in vegetative cells becoming heterocysts (7, 33, 34). The accumulation of active HetR in proheterocysts seems to trigger the downstream cascade. Once heterocyst differentiation is induced by nitrogen deficiency, the process can be halted if combined nitrogen is again available, as long as the so-called commitment point has not been reached.

A computational analysis (22) identified *alr3234*, a homolog of *hetP* described as a negative modulator of heterocyst commitment (14), as a potential target of NsiR1.4. We have confirmed the interaction between NsiR1.4 and *alr3234* mRNA by coexpression in E. coli (Fig. 1) and *in vitro* footprinting (Fig. 2). NsiR1.4 binds to a region that includes the start codon of *alr3234* and, thus, could inhibit translation initiation by hampering ribosome access. We have also confirmed the posttranscriptional regulation exerted by NsiR1.4 on *alr3234* expression in Nostoc (Fig. 3). A control strain (OE_C/GFP) that expressed the reporter *alr3234*::sf*gfp* in all cells of the filament showed a decrease in green fluorescence only in heterocysts. This negative regulation was observed even in cells at a very early stage of differentiation that still showed red autofluorescence, indicative of immature heterocysts (Fig. 3D). These observations would be in line with a negative regulation exerted by a heterocyst-specific sRNA, such as NsiR1, one of the earliest markers of cell differentiation in *Nostoc* sp. PCC 7120 (26). In fact, Western blot analysis (Fig. 3C) confirmed that the strain overexpressing NsiR1.4 showed a significant decrease in the accumulation of Alr3234::sfGFP (Fig. 3C). One of the phenotypic characteristics described for an *alr3234* mutant is an earlier commitment to heterocyst differentiation (14). Although the expected effects are not as drastic as those described for an *alr3234* mutant, the timing of commitment in strain OE_NsiR1.4 was, in fact, shifted to an earlier time point (about 3 h earlier than in the control strain OE_C), while OE_as_NsiR1, depleted of NsiR1, had a slight delay in commitment (Fig. 4B), consistent with an inhibitory effect of NsiR1.4 on *alr3234*. The putative positive effect of NsiR1.4 on heterocyst commitment is also in line with the observation that, in a solid medium, OE_NsiR1.4 differentiates heterocysts even in the presence of nitrate, a condition that suppresses heterocyst development in the wild type (Fig. S1). Overexpression of NsiR1 has been shown to result in an increased amount of HetR (27), and overexpression of HetR can induce heterocyst differentiation in the presence of combined nitrogen (35). Therefore, the observation that overexpression of NsiR1.4 leads to differentiation of heterocysts in the presence of nitrate could be due to increased accumulation of HetR. However, the strain overexpressing NsiR1.1, which accumulates larger amounts of HetR than the strain that overexpresses NsiR1.4 (27), does not differentiate heterocysts in the presence of nitrate. This suggests that the observed phenotype is not a consequence of increased amounts of HetR but is due instead to a specific effect of NsiR1.4, such as the release of the brake on differentiation resulting from a reduced amount of Alr3234 in cells overexpressing NsiR1.4.

Alr3234 represents a brake that delays commitment, providing a lag time to avoid unnecessary differentiation under short-term variations in combined nitrogen abundance (14). We hypothesize that only when enough NsiR1 has accumulated as a consequence of persistent nitrogen deficiency is the amount of Alr3234 low enough to release the brake on commitment. This mechanism would have a similar function to the described role of cyanobacterial AbrB proteins as safety devices that prevent heterocyst differentiation under fluctuating conditions (36).

Despite the slight difference in sequences between NsiR1.1 and NsiR1.4 (Fig. 1B), strains OE_NsiR1.1 and OE_NsiR1.4 had different phenotypes. They grew differentially in solid media with different nitrogen sources (27), and only OE_NsiR1.4/GFP had reduced amounts of Alr3234::sfGFP (Fig. 3C). Thus, these results suggest a divergence in the functions of NsiR1.1 and NsiR1.4, which could be explained by their different quantitative effects on *hetF* (27) and *alr3234* or by interactions with additional unknown targets specific for each of them. The positive effect of NsiR1.4 on differentiation is in accordance with the observation that OE_as_NsiR1, depleted of NsiR1, was delayed in differentiation, as indicated by delayed expression of *nifHDK* genes (27) and slightly delayed commitment (Fig. 4B).

In this work, we describe how different versions of one sRNA regulate different targets. Other instances of sRNAs encoded by multiple repeats have been described in diverse bacteria, including E. coli, Pseudomonas aeruginosa, Bacillus subtilis, and Sinorhizobium meliloti (see reference 37 for a review). In most cases, there is a regulatory redundancy, and the different copies of a given sRNA act additively to regulate one particular target. In other cases, their modes of action are nonredundant, regulating different targets. NsiR1.1 on one side and the identical copies NsiR1.3 to NsiR1.9 on the other represent divergent versions of an sRNA with partially redundant targets, *hetF* and *alr3234*, but possibly also additional targets. In fact, the different phenotypes observed for strains overexpressing NsiR1.1 or NsiR1.4 point to functional diversification in the case of multiple copies of NsiR1. In any case, the local expression of NsiR1 in proheterocysts would have a positive effect in the completion of differentiation of these particular cells.

**FIG 5:**
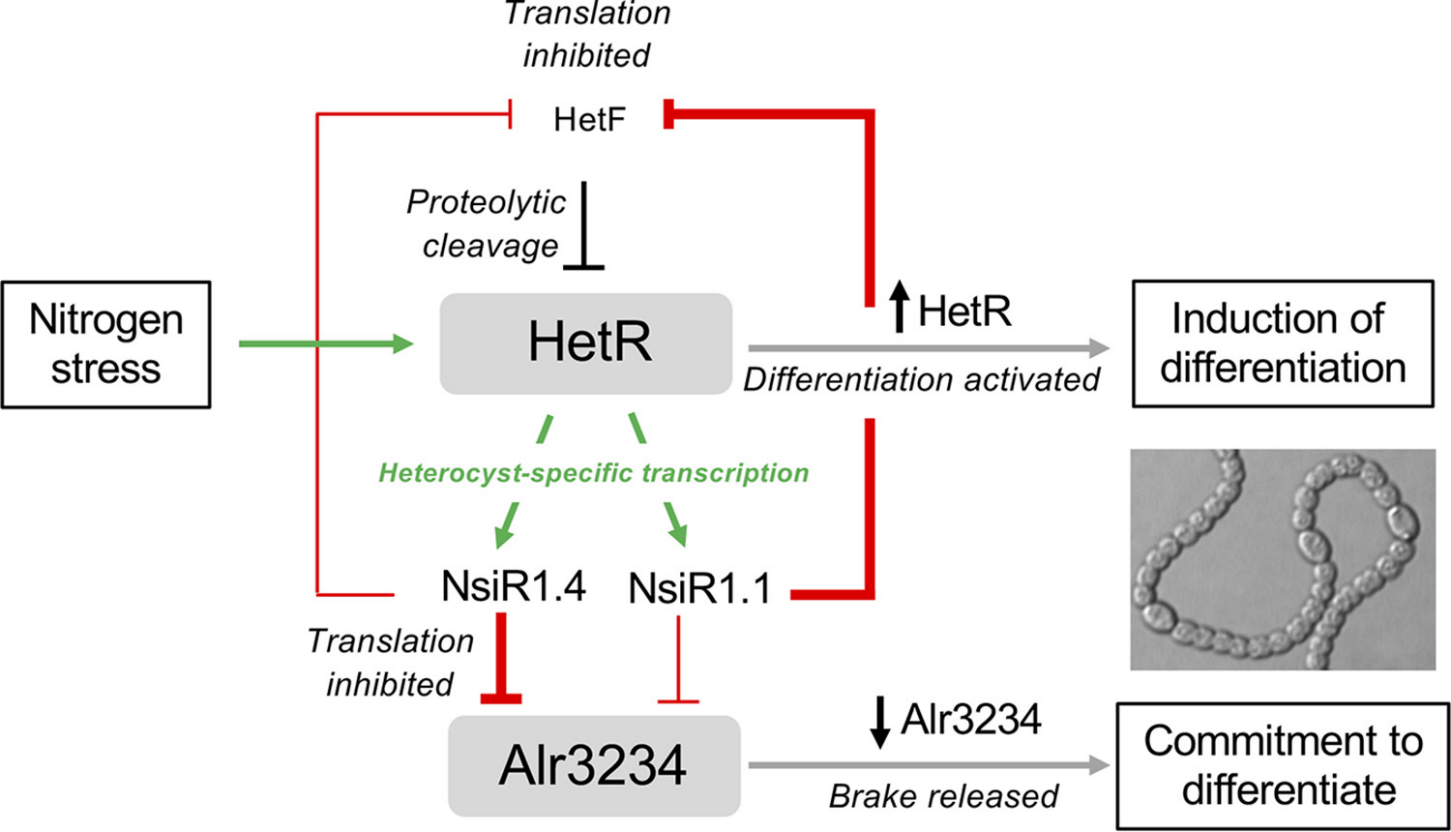
Model of the functions of NsiR1.1 and NsiR1.4 in the regulation of heterocyst differentiation and commitment. Events taking place specifically in cells developing as heterocysts are shown. Positive transcriptional regulation is indicated by green arrows. Negative posttranscriptional regulation by NsiR1 is indicated by red lines with blunt ends. Thin red lines indicate weaker regulation than thick red lines.

The current models of events leading to heterocyst differentiation and patterning are based on transcriptional regulation and protein-protein interactions. Beyond those mechanisms, we here identify a role for an sRNA that specifically accumulates in heterocysts. We propose that, even though *hetF* and *alr3234* are constitutively transcribed upon nitrogen deprivation, the early expression of NsiR1.1 and NsiR1.4 specifically in proheterocysts would provoke the posttranscriptional repression of *alr3234* in addition to repression of *hetF*, resulting in a local accumulation of HetR in proheterocysts on one side (triggering the downstream cascade of heterocyst differentiation) and releasing the brake exerted by Alr3234 on the commitment of proheterocysts to differentiate on the other (Fig. 5).

In addition to NsiR1, described here, other noncoding RNAs that are transcribed exclusively in heterocysts, such as the antisense to *glpX* (38), or at least more strongly in heterocysts than in adjacent vegetative cells, such as NsiR3 (39) or NsiR4 (40), contribute to the metabolic reprogramming that takes place in these specialized cells. So, the specific posttranscriptional regulation exerted by heterocyst-specific noncoding RNAs in the multicellular cyanobacterium Nostoc sp. PCC 7120 would be a good example of how complexity can emerge from the superposition of simple patterns, such as constitutive transcription of a given gene (e.g., *hetF* or *alr3234*) plus cell type-specific negative posttranscriptional regulation mediated by a noncoding RNA (NsiR1 in this case). Further study of additional potential targets for NsiR1 and the characterization of additional heterocyst-specific noncoding RNAs (23) could reveal the importance of these molecules in the regulation of this developmental process.

## MATERIALS AND METHODS

### Strains and growth conditions.

For Western blot analysis, cultures of different derivative strains of Nostoc sp. PCC 7120 (Table S1) were grown for 1 week in flasks at 30°C in liquid BG11 medium (41) lacking NaNO_3_ but containing 3 mM NH_4_Cl and 6 mM *N*-tris(hydroxymethyl)methyl-2-aminoethanesulfonic acid–NaOH buffer (pH 7.5) (BG11_0_ plus 3 mM NH_4_Cl). Nitrogen deficiency was induced by filtering and washing cells and resuspending them in nitrogen-free medium (BG11_0_). The chlorophyll concentration of the cultures was measured as described previously (42). The derivative strains of *Nostoc* were grown in the presence of streptomycin (Sm) and spectinomycin (Sp) at 2 μg/mL each (liquid medium) or 5 μg/mL each (solid medium). Escherichia coli strains were grown in LB medium supplemented with appropriate antibiotics (43).

### Reporter assay for *in vivo* verification of targets.

We used the GFP reporter system described in reference 31 with superfolder GFP (sfGFP) and plasmid pXG10-SF (44) for the experimental verification of targets in E. coli. The whole intergenic region of *alr3233* to *alr3234* plus 60 nucleotides within the *alr3234* coding region, containing the predicted NsiR1 interaction sequence, was amplified using oligonucleotides 553 and 870. The PCR product was digested with NsiI-NheI and cloned into NsiI-NheI-digested vector pXG10-SF, resulting in translational fusions of truncated Alr3234 to sfGFP (plasmid pMBA86) (Table S2). To express NsiR1.1 and NsiR1.4 in E. coli, plasmids pMML3 and pMML4 (27) were used. For the mutagenesis of the *alr3234* 5′ UTR, a plasmid similar to pMBA86 was constructed using, in addition to the oligonucleotides mentioned, overlapping PCR with primers 871 and 872 containing the desired changes (pMBA87, Table S2) (see Table S3 for oligonucleotides information). The positions for the mutations of the *alr3234* 5′ UTR were selected based on the hybridization energies predicted by IntaRNA (30). Different combinations of plasmids were introduced into E. coli DH5α, and GFP expression was determined by Western blot analysis. The sequences of inserts from plasmids used in the heterologous reporter system are shown in Table S4.

### Construction of *Nostoc* sp. PCC 7120-derived strains.

We used pMBA51 (38), pMBA42, pMBA77, and pMBA78 (27) as backbones for the coexpression of different versions of NsiR1 from the *trc* promoter and the 5′ UTR of *alr3234* plus 60 nucleotides of its coding sequence fused to sf*gfp* from the *rnpB* promoter (Table S2). The *rnpB* promoter was amplified from pMBA20 (38) using oligonucleotides 501 and 938 (PCR A). The 5′ UTR of *alr3234* fused to sf*gfp* was amplified from pMBA86 using oligonucleotides 939 and 940 (PCR B). An overlapping fragment was amplified using as the templates PCR A and B and oligonucleotides 501 and 940. The resulting PCR product was digested with ClaI and PstI and cloned in ClaI-PstI-digested pMBA51, pMBA77, pMBA78, and pMBA42, rendering, respectively, pMBA96, pMBA97, pMBA98, and pMBA99 (see Table S2 for plasmid descriptions). pMBA96, pMBA97, pMBA98, and pMBA99 were introduced into *Nostoc* sp. PCC 7120 by conjugation (45).

### Fluorescence microscopy.

The fluorescence of Nostoc sp. PCC 7120 filaments carrying plasmid pMBA96 or pMBA99 (Table S2) growing on top of solidified nitrogen-free medium was analyzed and quantified as described previously (26) using a Leica HCX PLAN-APO 63× 1.4 numeric aperture (NA) oil immersion objective attached to a Leica TCS SP2 laser scanning confocal microscope. Samples were excited at 488 nm by an argon ion laser, and fluorescent emissions were monitored by collection across windows of 500 to 538 nm (GFP) and 630 to 700 nm (autofluorescence of photosynthetic pigments). The expression of Alr3234::GFP in heterocysts and vegetative cells was quantified in regions of interest (ROI) of images corresponding to heterocysts and their two adjacent vegetative cells (see scheme in Fig. 3E).

### *In vitro* synthesis and labeling of RNA.

RNA transcripts were generated *in vitro* with the MEGAscript high-yield transcription kit (catalog number AM1333; Thermo Fisher Scientific). The DNA template for the transcription of the *alr3234* 5′ UTR (wild type or MUT variant), NsiR1.1, or NsiR1.4 was generated by PCR using a primer that included a T7 promoter sequence upstream from the 5′ end of the RNA and a primer that matched the 3′ end of the RNA (Table S3). The templates used for these PCR amplifications were pMBA86 for the *alr3234* 5′ UTR and pMBA87 for the *alr3234* (MUT) 5′ UTR or the overlapping oligonucleotides for NsiR1.1 and NsiR1.4. The sequences of the RNAs transcribed *in vitro* are shown in Table S5. Three (*alr3234*) or one (*nsiR1*) non-encoded guanosine were added at the 5′ end of each template for efficient T7 transcription. After *in vitro* transcription, the RNAs were treated with DNase I and purified by phenol, phenol-chloroform, and chloroform extractions, precipitated with ethanol at −20°C, and washed with 70% ethanol. 100 pmol of RNAs were labeled at the 5′ end with [γ-^32^P]ATP and polynucleotide kinase and purified in a 6% polyacrylamide gel as described previously (46).

### *In vitro* footprinting.

RNase T1 and lead(II) acetate were used for structure probing with *in vitro*-transcribed RNAs (Table S5) as described previously (46). For the footprinting of NsiR1.1 or NsiR1.4 on the *alr3234* 5′ UTR, 0.1 pmol of labeled *alr3234* 5′ UTR RNAs (wild type or MUT variant) were mixed in 7-μl volumes with water (−) or 1 pmol of NsiR1.1 or NsiR1.4 (+), denatured for 1 min at 95°C, and chilled on ice for 5 min. After the denaturing and chilling steps, we added 1 μl of 1-mg/mL yeast RNA (Thermo Fisher Scientific AM7118) and 1 μl of 10× structure buffer (Thermo Fisher Scientific). The samples were incubated for 15 min at 37°C. Treatments with RNase T1 and lead(II) were performed as described previously (46). Alkaline and RNase T1 G ladders were generated as described previously (46). All samples were run on 6% polyacrylamide, 7 M urea gels, and bands visualized with a Cyclone storage phosphor system. The bands were quantified with ImageQuant TL software (Cytiva).

### Heterocyst differentiation and commitment assay.

To test heterocyst commitment, cells from strains OE_C, OE_NsiR1.4, and OE_as_NsiR1 were taken from plates containing nitrate and grown in 100 mL of liquid BG11_0_ plus 4 mM NH_4_Cl for 72 h before being subjected to nitrogen deficiency. Nitrogen deficiency was induced by filtration and washing of the cells as described above. Washed cells were resuspended in 100 mL of nitrogen-free BG11_0_ medium in a 250-mL Erlenmeyer flask and incubated at 30°C with shaking. At different times, 2-mL amounts were transferred to a glass tube, supplemented with 4 mM NH_4_Cl, and further incubated for 24 to 36 h prior to heterocyst counting (Fig. 4A). Heterocysts were stained with Alcian blue as described previously (47). The frequency of heterocysts was calculated as the number of cells stained by Alcian blue per at least 500 individual cells counted. All results are expressed as the average of 2 replicates ± standard deviation (SD).

### Nitrate and nitrite reductase assay.

Cultures of strains OE_C and OE_NsiR1.4 were grown photoautotrophically at 30°C and bubbled with an air/CO_2_ mixture (1%, vol/vol) in BG11_0_C (BG11_0_ medium with 10 mM NaHCO_3_) plus 6 mM NH_4_Cl. Exponentially growing cells were harvested by filtration, washed with BG11_0_C, and resuspended in media containing three different nitrogen sources. Aliquots of 20 mL at 4 μg chlorophyll/mL were incubated at 30°C in BG11_0_C (N_2_), BG11C (NO_3_^−^), or BG11_0_C plus 6 mM NH_4_Cl (NH_4_^+^) bubbled with an air/CO_2_ mixture (1%, vol/vol). After 4 h of incubation, cells were concentrated by centrifugation to a final concentration of 50 μg chlorophyll/mL. Nitrate reductase (48) and nitrite reductase (49) assays were performed with numbers of cells corresponding to 5 and 25 μg chlorophyll, respectively. Dithionite-reduced methyl viologen was used as the reductant in cells made permeable with mixed alkyltrimethylammonium bromide.

### Western blot analysis.

For Alr3234::GFP Western blot analysis in E. coli, cells from stationary-phase cultures were harvested and resuspended in SDS-PAGE loading buffer. Proteins were fractionated in 10% SDS–PAGE. Antibodies against GFP (Roche) and E. coli GroEL (Sigma-Aldrich) were used. For Alr3234::GFP Western blot analysis in *Nostoc*, soluble fractions were prepared as previously described (40). Forty-microgram amounts of protein were fractionated on 10% SDS–PAGE gels, and antibodies against GFP were used. Ponceau staining was used as a loading control. The ECL plus immunoblotting system (GE Healthcare) was used to detect the different primary antibodies using anti-rabbit (Sigma-Aldrich) or anti-mouse (Bio-Rad) horseradish peroxidase-conjugated secondary antibodies.

### RNA isolation, Northern blot analysis, and primer extension assays.

Total RNA was isolated using hot phenol as described previously (50), with modifications (24). The samples (6 μg of total RNA) were separated in 8% urea–polyacrylamide gels as described previously (51) and transferred to Hybond-N+ membrane (Amersham). Oligonucleotides 987 and 963 were end labeled with [γ-^32^P]ATP and polynucleotide kinase and used as probes for NsiR1.1 and NsiR1.4, respectively.

### Statistical methods.

Student’s *t* test was used to determine statistical significance. The numbers of biological samples can be found in the figure legends.
