# Supplementary material for: The Heterocyst-Specific Small RNA NsiR1 Regulates the Commitment to Differentiation in Nostoc
Source: Microbiol Spectr. 2022 Mar 1;10(2):e02274-21. doi: 10.1128/spectrum.02274-21 (PMC9045159; doi:10.1128/spectrum.02274-21)
Supplement: SUPPLEMENTAL FILE 1 — Supplemental material. Download SPECTRUM02274-21_Supp_1_seq9.pdf, PDF file, 0.4 MB [file spectrum02274-21_supp_1_seq9.pdf]

Supplemental Materials for:

The heterocyst-specific sRNA NsiR1 regulates the commitment to differentiation in *Nostoc*

Manuel Brenes-Álvarez, Agustín Vioque, Alicia M. Muro-Pastor

This PDF includes:

-Supplemental Figure S1

-Supplemental Tables S1 to S5

-References for supplemental material citations

Figure S1

A)

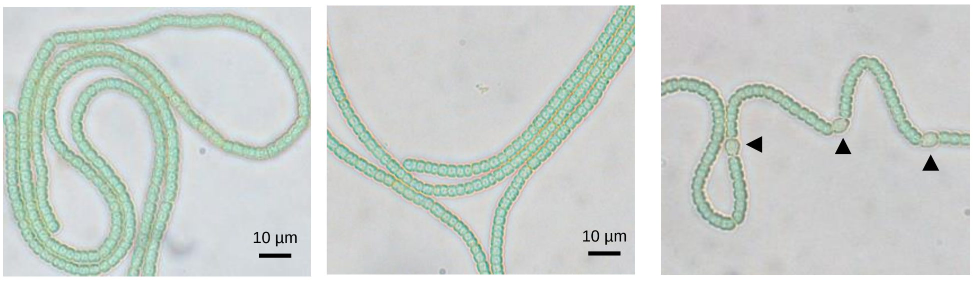

OE_C **OE_NsiR1.1** **OE_NsiR1.4**

B)

	Nitrate reductase (mU/mg)			Nitrite reductase (mU/mg)		
Strain	NH_4^+	NO_3^-	N_2	NH_4^+	NO_3^-	N_2
OE_C	1.94 ± 0.46	19.41 ± 0.41	2.93 ± 0.23	ND	5.61 ± 1.08	ND
OE_NsiR1.4	1.95 ± 0.11	10.04 ± 0.19	2.43 ± 0.03	ND	2.68 ± 0.14	ND

Figure S1. Strain overexpressing NsiR1.4 differentiates heterocysts in the presence of nitrate. (A) Bright-field images of filaments of OE_C, OE_NsiR1.1, and OE_NsiR1.4 strains growing on solid media containing nitrate. Black triangles point to heterocysts. (B) Nitrate and nitrite reductase activities. The data are given as the mean ± standard deviation of nitrate or nitrite reductase specific activity (mU/mg total protein) of three independent clones of the OE_C strain and two independent clones of OE_NsiR1.4 in the presence of different nitrogen sources; ND, not determined.

Table S1. Strains

Strain	Description	Reference
Escherichia coli		
DH5α	Used for routine transformation	(1)
***Nostoc* sp.**		
PCC 7120	Wild type	Pasteur Culture Collection
OE_C	Sm^RSp^R, pMBA51 inserted in plasmid alpha. *T1* terminator of *E. coli rrnB* gene expressed constitutively from the *trc* promoter.	(2)
OE_NsiR1.1	Sm^RSp^R, pMBA77 inserted in plasmid alpha. NsiR1.1 transcribed constitutively from the *trc* promoter. Overexpression of NsiR1.1.	(3)
OE_NsiR1.4	Sm^RSp^R, pMBA78 inserted in plasmid alpha. NsiR1.4 expressed constitutively from *trc* promoter. Overexpression of NsiR1.4.	(3)
OE_as_NsiR1	Sm^RSp^R, pMBA42 inserted in plasmid alpha. Antisense to NsiR1 transcribed constitutively from the *trc* promoter. Overexpression of an antisense to NsiR1 resulting in depletion of NsiR1.	(3)
OE_C/GFP	Sm^RSp^R, pMBA96 inserted in plasmid alpha. *T1* terminator of *E. coli rrnB* gene expressed constitutively from the *trc* promoter and 5' UTR of *alr3234* plus 60 nucleotides of its coding sequence fused to sf*gfp* expressed from the *Nostoc rnpB* promoter.	This work
OE_NsiR1.1/GFP	Sm^RSp^R, pMBA97 inserted in plasmid alpha. NsiR1.1 transcribed constitutively from the *trc* promoter and 5' UTR of *alr3234* plus 60 nucleotides of its coding sequence fused to sf*gfp* expressed from the *Nostoc rnpB* promoter.	This work
OE_NsiR1.4/GFP	Sm^RSp^R, pMBA98 inserted in plasmid alpha. NsiR1.4 transcribed constitutively from the *trc* promoter and 5' UTR of *alr3234* plus 60 nucleotides of its coding sequence fused to sf*gfp* expressed from the *Nostoc rnpB* promoter.	This work

OE_as_NsiR1/GFP	Sm^RSp^R, pMBA99 inserted in plasmid alpha. Antisense to NsiR1 transcribed constitutively from the *trc* promoter and 5' UTR of *alr3234* plus 60 nucleotides of its coding sequence fused to sf*gfp* expressed from the *Nostoc rnpB* promoter.	This work

Table S2. Plasmids

Name	Description	Reference
pJV300	Control plasmid expressing a ~50 nt transcript derived from the *T1* terminator of *E. coli rrnB* gene.	(4)
pXG0	Cm^R, control plasmid without GFP	(5)
pXG10-SF	Cm^R, plasmid for construction of translational sfGFP fusions expressed from P_{LtetO} promoter.	(6)
pZE12-luc	Ap^R, plasmid used for the expression of NsiR1 from P_{LlacO} promoter.	(7)
pMML3	Ap^R, plasmid based on pZE12-luc expressing NsiR1.1.	(3)
pMML4	Ap^R, plasmid based on pZE12-luc expressing NsiR1.4.	(3)
pMBA20	$Ap^RSm^RSp^R$, plasmid for the overexpression of transcripts from the rnpB promoter and followed by the *T1* transcriptional terminator of *E. coli rrnB* gene.	(2)
pMBA42	$Ap^RSm^RSp^R$, plasmid for the expression of an antisense to NsiR1 from *trc* promoter.	(3)
pMBA51	$Ap^RSm^RSp^R$, control plasmid, expresses a 56 nt transcript derived from the *T1* terminator of *E. coli rrnB* gene from the *trc* promoter.	(2)
pMBA77	$Ap^RSm^RSp^R$, plasmid for the overexpression NsiR1.1 from *trc* promoter.	(3)
pMBA78	$Ap^RSm^RSp^R$, plasmid for the overexpression NsiR1.4 from *trc* promoter.	(3)
pMBA86	Cm^R, sfGFP reporter plasmid based on pXG10-SF containing the *alr3234* 5'UTR plus sequences encoding the first 20 amino acids of Alr3234.	This work
pMBA87	Cm^R, same as pMBA86 but with T4G and G9A substitutions in the *alr3234* 5'UTR (*alr3234* MUT).	This work
pMBA96	$Ap^RSm^RSp^R$, control plasmid, based on pMBA51, expresses a 56 nt transcript derived from the *T1* terminator of *E. coli rrnB* gene from the *trc* promoter and a translational fusion of *alr3234* 5'UTR plus sequences encoding the first 20 amino acids of Alr3234 fused to GFP from the *Nostoc rnpB* promoter.	This work
pMBA97	$Ap^RSm^RSp^R$, based on pMBA77, plasmid for the overexpression of NsiR1.1 from the *trc* promoter and a translational fusion of *alr3234* 5'UTR plus sequences encoding the first 20 amino acids of Alr3234 fused to GFP from the *Nostoc rnpB* promoter.	This work

pMBA98	$Ap^R Sm^R Sp^R$, based on pMBA78, plasmid for the overexpression of NsiR1.4 from the *trc* promoter and a translational fusion of *alr3234* 5'UTR plus sequences encoding the first 20 amino acids of Alr3234 fused to GFP from the *Nostoc rnpB* promoter.	This work
pMBA99	$Ap^R Sm^R Sp^R$, based on pMBA42, plasmid for the overexpression of an antisense to NsiR1 from the *trc* promoter and a translational fusion of *alr3234* 5'UTR plus sequences encoding the first 20 amino acids of Alr3234 fused to GFP from the *Nostoc rnpB* promoter.	This work

Table S3. Oligonucleotides

Name	Sequence (5'-3')	Used for
553	GTTTTATGCATACAATCATTAAAAATGTGGTGCAG	Cloning of *alr3234* 5'UTR in pXG10-SF
870	GTTTTGCTAGCTACTTGCTCATTATTTATTTTC	
871	AATGTCTTCTTGATCCATAACTTCCCCTGAT	Mutagenesis of *alr3234* 5'UTR
872	AAGTTATGGATCAAGAAGACATTTACAATTC	
833	**TAATACGACTCACTATAG**GGTAGATGCACCTTGATCTTTAACTCCCCTAGTTGGTTG	*In vitro* transcription from the T7 promoter of NsiR1.1
834	AAAAAACCCCCAGTGGCTATCAACCAACTAGGGGAGTTAAAGATC	
835	**TAATACGACTCACTATAG**GGTAGATGCACCCTGATAACTAACTCCCCTAGCTGGCTAAC	*In vitro* transcription from the T7 promoter of NsiR1.4
836	AAAAAAGCCCCCAGTCGGTGTTAGCCAGCTAGGGGAGTTAG	
878	**TAATACGACTCACTATAGGG**ACAATCATTAAAAATGTGGTGCAG	*In vitro* transcription from the T7 promoter of *alr3234* 5'UTR
879	TACTTGCTCATTATTTATTTTCTTAACG	
501	GTTTTATCGATGTCCCTTCACTATCAAAAAAC	Cloning of *alr3234* 5'UTR plus sequences encoding the first 20 amino acids of Alr3234 fused to sfGFP (transcribed from the *rnpB* promoter)
938	TTGTAGTACTGCTCCAGTGTAATCTTCGTTGG	
939	GAGCAGTACTACAATCATTAAAAATGTGGTGCAG	
940	GTTTTCTGCAGAGGGCGGCGGATTTGT	

Table S4. Sequences of inserts in plasmids used for verification of sRNA-mRNA interactions in *E. coli*.

Plasmid	Sequence	Description
pMBA86	atgcatACAATCATTAAAAATGTGGTGCAGACATATACCCAAACTT<br>TAGAAACATCAATTTACTCTTAGTGGTAATGGGTAATATAGCTGTT<br>GACAGTTAACTGTTGACAGTTGACTGTTGACAGATTTGAAAGTCTT<br>GTTTTATCAGGGTTTGATGTTAGCTGGATGTTTTAATCTATCTGGC<br>TACAGCTATAAATGTAATTCTCTTCTCCATCACCAATTACCAATTA<br>CCAATTACCAATTTTCAAACCATCAAATAAAATCATCAGGGGAAGT<br>T**ATG**TATCAGGAAGACATTTACAATTCACAGAACGTTAAGAAAATA<br>AATAATGAGCAAGTAgctagc	*alr3234* 5'UTR
pMBA87	atgcatACAATCATTAAAAATGTGGTGCAGACATATACCCAAACTT<br>TAGAAACATCAATTTACTCTTAGTGGTAATGGGTAATATAGCTGTT<br>GACAGTTAACTGTTGACAGTTGACTGTTGACAGATTTGAAAGTCTT<br>GTTTTATCAGGGTTTGATGTTAGCTGGATGTTTTAATCTATCTGGC<br>TACAGCTATAAATGTAATTCTCTTCTCCATCACCAATTACCAATTA<br>CCAATTACCAATTTTCAAACCATCAAATAAAATCATCAGGGGAAGT<br>T**ATG**GATCAAGAAGACATTTACAATTCACAGAACGTTAAGAAAATA<br>AATAATGAGCAAGTAgctagc	*alr3234* (MUT) 5'UTR
pMML3	GGTAGATGCACCTTGATCTTTAACTCCCCTAGTTGGTTGATAGCCA<br>CTGGGGGTTTTTTCTATAACTAGCATATACTTAGATTATTCTACTG<br>TCTTGGGtctaga	NsiR1.1
pMML4	GGTAGATGCACCCTGATAACTAACTCCCCTAGCTGGCTAACACCGA<br>CTGGGGGCTTTTTTATGTTCATAAATAACCAGCATCATTGTGCAGA<br>TTCATCCGTCAGATCTAGAtctaga	NsiR1.4

Nostoc sequences are capitalized, with black letters corresponding to 5'UTR and blue letters corresponding to coding sequences, respectively. NsiI, NheI and XbaI sites that were used for cloning are highlighted in magenta, yellow or blue, respectively. Nucleotide changes with respect to wild type sequences are indicated in red. Grey shadowed letters indicate the NsiR1.1 and NsiR1.4 sequences. Start codons are shown in bold and underlined.

Table S5. RNAs used for *in vitro* footprinting assays.

Name	Sequence (5'-3')
NsiR1.1	gGGUAGAUGCACCUUGAUCUUUAACUCCCCUAGUUGGUUGAUAGCCACUGGGGG UUUUUU
NsiR1.4	gGGUAGAUGCACCCUGAUAACUAACUCCCCUAGCUGGCUAACACCGACUGGGGG CUUUUUU
alr3234 5'UTR	gggACAAUCAUUAAAAAUGUGGUGCAGACAUAUACCCAAACUUUAGAAACAUCA AUUUACUCUUAGUGGUAAUGGGUAAUAUAGCUGUUGACAGUUAACUGUUGACAG UUGACUGUUGACAGAUUUGAAAGUCUUGUUUUAUCAGGGUUUGAUGUUAGCUGG AUGUUUUAAUCUAUCUGGCUACAGCUAUAAAUGUAAUUCUCUUCUCCAUCACCA AUUACCAAUUACCAAUUACCAAUUUUCAAACCAUCAAAUAAAAUCAUCAGGGGA AGUU**AUG**UAUCAGGAAGACAUUUACAAUUCACAGAACGUUAAGAAAAUAAAUAA UGAGCAAGUA
alr3324 (MUT) 5'UTR	gggACAAUCAUUAAAAAUGUGGUGCAGACAUAUACCCAAACUUUAGAAACAUCA AUUUACUCUUAGUGGUAAUGGGUAAUAUAGCUGUUGACAGUUAACUGUUGACAG UUGACUGUUGACAGAUUUGAAAGUCUUGUUUUAUCAGGGUUUGAUGUUAGCUGG AUGUUUUAAUCUAUCUGGCUACAGCUAUAAAUGUAAUUCUCUUCUCCAUCACCA AUUACCAAUUACCAAUUACCAAUUUUCAAACCAUCAAAUAAAAUCAUCAGGGGA AGUU**AUG**GAUCAAGAAGACAUUUACAAUUCACAGAACGUUAAGAAAAUAAAUAA UGAGCAAGUA

Nostoc sequences are capitalized, guanosines added for T7 transcription efficiency are in lower case. Predicted interaction sites of the two versions of *alr3234* 5'UTR with different versions of NsiR1 are highlighted in yellow. Start codons are shown in bold and underlined. Mutations introduced in *alr3234* (MUT) 5'UTR are indicated in red.

Supplemental References:

1. **Hanahan D.** 1983. Studies on transformation of *Escherichia coli* with plasmids. J Mol Biol **166:**557-580.
2. **Olmedo-Verd E, Brenes-Álvarez M, Vioque A, Muro-Pastor AM.** 2019. A heterocyst-specific antisense RNA contributes to metabolic reprogramming in *Nostoc* sp. PCC 7120. Plant Cell Physiol **60:**1646-1655.
3. **Brenes-Álvarez M, Minguet M, Vioque A, Muro-Pastor AM.** 2020. NsiR1, a small RNA with multiple copies, modulates heterocyst differentiation in the cyanobacterium *Nostoc* sp. PCC 7120. Environ Microbiol **22:**3325-3338.
4. **Sittka A, Pfeiffer V, Tedin K, Vogel J.** 2007. The RNA chaperone Hfq is essential for the virulence of *Salmonella typhimurium*. Mol Microbiol **63:**193-217.
5. **Urban JH, Vogel J.** 2007. Translational control and target recognition by *Escherichia coli* small RNAs in vivo. Nucleic Acids Res **35:**1018-1037.
6. **Corcoran CP, Podkaminski D, Papenfort K, Urban JH, Hinton JC, Vogel J.** 2012. Superfolder GFP reporters validate diverse new mRNA targets of the classic porin regulator, MicF RNA. Mol Microbiol **84:**428-445.
7. **Lutz R, Bujard H.** 1997. Independent and tight regulation of transcriptional units in *Escherichia coli* via the LacR/O, the TetR/O and AraC/I1-I2 regulatory elements. Nucleic Acids Res **25:**1203-1210.
